# Identification and structural analysis of C-terminally truncated collapsin response mediator protein-2 in a murine model of prion diseases

**DOI:** 10.1186/1477-5956-8-53

**Published:** 2010-10-20

**Authors:** Fumiko Shinkai-Ouchi, Yoshio Yamakawa, Hideyuki Hara, Minoru Tobiume, Masahiro Nishijima, Kentaro Hanada, Ken'ichi Hagiwara

**Affiliations:** 1 Department of Biochemistry and Cell Biology, National Institute of Infectious Diseases, 1-23-1, Toyama, Shinjuku-ku, Tokyo 162-8640, Japan; 2 Department of Pathology, National Institute of Infectious Diseases, 1-23-1, Toyama, Shinjuku-ku, Tokyo 162-8640, Japan; 3 National Institute of Health Sciences, Kamiyoga, Setagaya-ku, Tokyo 158-8501, Japan

## Abstract

**Background:**

Prion diseases are fatal neurodegenerative disorders that accompany an accumulation of the disease-associated form(s) of prion protein (PrP^Sc^) in the central nervous system. The neuropathological changes in the brain begin with focal deposits of PrP^Sc^, followed by pathomorphological abnormalities of axon terminal degeneration, synaptic loss, atrophy of dendritic trees, and eventual neuronal cell death in the lesions. However, the underlying molecular basis for these neuropathogenic abnormalities is not fully understood.

**Results:**

In a proteomic analysis of soluble proteins in the brains of mice challenged intracerebrally with scrapie prion (Obihiro I strain), we found that the amount of the full-length form of collapsin response mediator protein-2 (CRMP-2; 61 kDa) decreased in the late stages of the disease, while the amount of its truncated form (56 kDa) increased to comparable levels observed for the full-length form. Detailed analysis by liquid chromatography-electrospray ionization-tandem mass spectrometry showed that the 56-kDa form (named CRMP-2-ΔC) lacked the sequence from serine^518 ^to the C-terminus, including the C-terminal phosphorylation sites important for the regulation of axonal growth and axon-dendrite specification in developing neurons. The invariable size of the mRNA transcript in Northern blot analysis suggested that the truncation was due to post-translational proteolysis. By overexpression of CRMP-2-ΔC in primary cultured neurons, we observed the augmentation of the development of neurite branch tips to the same levels as for CRMP-2^T514A/T555A^, a non-phosphorylated mimic of the full-length protein. This suggests that the increased level of CRMP-2-ΔC in the brain modulates the integrity of neurons, and may be involved in the pathogenesis of the neuronal abnormalities observed in the late stages of the disease.

**Conclusions:**

We identified the presence of CRMP-2-ΔC in the brain of a murine model of prion disease. Of note, C-terminal truncations of CRMP-2 have been recently observed in models for neurodegenerative disorders such as ischemia, traumatic brain injury, and Wallerian degeneration. While the structural identity of CRMP-2-ΔC in those models remains unknown, the present study should provide clues to the molecular pathology of degenerating neurons in prion diseases in connection with other neurodegenerative disorders.

## Background

Transmissible spongiform encephalopathies, or prion diseases, are fatal neurodegenerative disorders that include Creutzfeldt-Jakob disease, Gerstmann-Sträussler-Scheinker disease, fatal familial insomnia and kuru in humans, scrapie in sheep and goats, and bovine spongiform encephalopathy in cattle. The diseases are characterized by the accumulation of the disease-associated form(s) of prion protein (PrP^Sc^) in the central nervous system and neuronal loss and vacuolation, although these features are not evident in some cases [1]. Analyses in murine models of scrapie showed that the neuropathological changes begin with the local deposition of PrP^Sc^, followed by axon terminal degeneration, synaptic loss, and atrophy of dendritic trees in the lesions [2,3]. PrP^Sc ^is a conformational isoform of the cellular prion protein (PrP^C^) encoded by the host *prnp *gene. While PrP^C ^is susceptible to digestion by proteinase K (PK), PrP^Sc ^is partially resistant to PK and is considered to be the infectious agent [1,4].

PrP^C ^is a glycosylphosphatidylinositol-anchored protein, and resides in the so-called lipid raft domains of the outer leaflets of plasma membranes, in endosomes, and in lysosomes [1]. An electron microscopic analysis showed the distribution of PrP^C ^on the plasma membranes of dendrites and spines, as well as in dendritic transport vesicles, endosomes, the axolemma, axonal transport vesicles and the myelin sheath [5]. Its physiological functions remain unknown, but it is proposed to be involved in cell-to-cell recognition, signal transduction by coupling with certain transmembrane-type receptors, response to oxidative stress, or the uptake of metal ions into cells [6]. Nevertheless, the observation that *prnp*^-/- ^mice are viable [7] indicates the functional redundancy of PrP^C^, and raises the question of whether a 'loss-of-function' of PrP^C ^is responsible for the neuronal cell death [8]. It is equally unclear whether PrP^Sc ^triggers neuronal cell death [8]. To understand the molecular neuropathology of prion diseases, microarray-based gene expression profiling has been conducted in murine models [9-13] and in cultured cells infected with the scrapie agent [14]. Proteomic analysis also identified 54 proteins differentially expressed in prion-infected cultured cells [15]. However, DNA microarray-based approaches [9-14] cannot detect the post-translational modifications of proteins, and the proteomic analysis in cultured cells [15] focused on the quantitative changes of proteins rather than their post-translational modifications.

In this study, we conducted a proteomic analysis of the brain in a murine model of scrapie to explore the molecular neuropathology of prion disease, and identified a truncated form of collapsin response mediator protein-2 (CRMP-2). Liquid chromatography-electrospray ionization-tandem mass spectrometry (LC-ESI-MS/MS) revealed that the truncation occurred on the carboxylic side of Ser^517^. CRMP-2, also known as CRMP-62, dihydropyrimidinase-related protein 2, Unc-33-like protein, or TOAD-64 [16-19], is a mediator of axonal outgrowth and axon-dendrite specification [16-20], and its activity is regulated through the sequential phosphorylation of its carboxyl-terminal (C-terminal) region by Cdk5 and GSK-3β or by Rho-kinase [20]. As the truncation at Ser^517 ^caused the partial ablation of the C-terminal phosphorylation sites, we further examined if the truncation affects the morphology of neurites in primary cultured neuronal cells.

## Results

### Two-dimensional electrophoresis of the soluble fractions from brain homogenates

**Figure 1:**
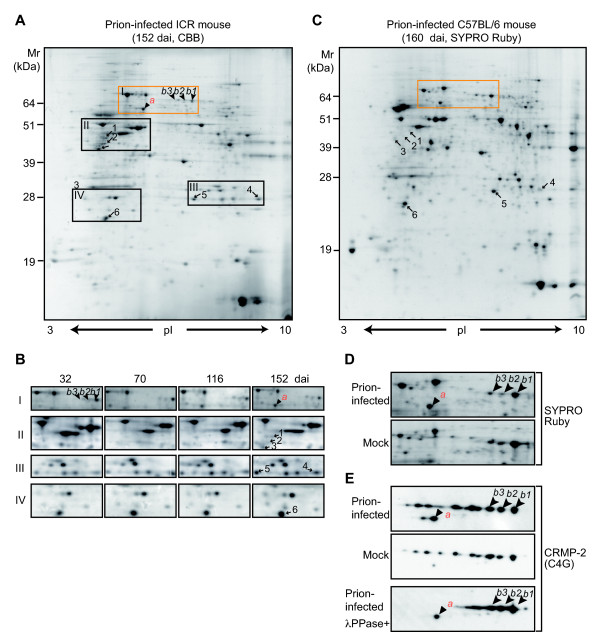
**Two-DE analysis of soluble brain fractions**. (A and B) CBB-stained gel obtained from a prion-infected ICR mouse (152 dai), and magnified images of boxes I-IV. The numbered spots differed in signal intensity between the prion-infected mice and the mock-infected controls, and were identified as listed in Table 1. (C) SYPRO Ruby-stained gel obtained from a prion-infected C57BL/6J mouse (160 dai). (D) The magnified image of the boxed area in (C) in comparison with the corresponding area of a 2-DE gel from a control C57BL/6J mouse. (E) Western blot analysis using the C4G anti-CRMP-2 antibody on the corresponding samples in (D). The lower panel shows the sample pre-incubated with λ-PPase.

Female C57BL/6J and ICR mice inoculated intracerebrally with prion (Obihiro-I strain) [21] began to show symptoms at approximately 130 days after inoculation (dai), fell ill by 170 dai, and were euthanized by 190 dai having entered the terminal stage. The accumulation of PrP^Sc ^in the brain became detectable by Western blotting at approximately 100 dai and was obvious from 130 dai (see below), as previously reported [22]. By contrast, the mock-infected control mice were healthy throughout the experiment. Using two-dimensional electrophoresis **(**2-DE) analysis and matrix-assisted laser desorption/ionization-mass spectrometry (MALDI-MS) (C57BL/6J sacrificed at 40, 69, 101, 133, and 160 dai, n = 2; ICR at 32, 70, 116, and 152 dai, n = 3), we found increases in the levels of glutathione S-transferase-μ1 and peroxiredoxin-6 (Figures 1A, B, and 1C, spots 4 and 5, respectively; Table 1). We also detected an increased abundance of glial fibrillary acidic protein (GFAP) in the infected mice (Figure 1B, spots 1-3; Table 1), as previously reported [9-11,13,23,24].

**Table 1:** Table 1 Summary of the mass spectrometry analysis

	2-DE^b)^		Mass spectrometry						Theoretical^b)^		Fold change^d)^(160dai)	_*p*_e)
					
**Spot**^**a)**^	**Mr**_**obs **_**(kDa)**	**pI**_**obs**_	Method	Identity	UniProt KB accession	Coverage	Number of peptides	**Mowse score**^**c)**^	**Mr**_**cal **_**(kDa)**	**pI**_**cal**_		
*a*	56	5.45	MALDI & LC-ESI	CRMP-2-ΔC	O08553	39	17	963	56.5^f)^	5.48^f)^	7.96	0.076
*b1*	61	5.77	MALDI & LC-ESI	CRMP-2	O08553	45	22	1419	62.3^g)^	5.95^g)^	0.53	0.375
*b2*	61	5.72	MALDI & LC-ESI	CRMP-2	O08553	8	4	61	62.3	5.95^h)^	0.39	0.249
*b3*	61	5.68	MALDI & LC-ESI	CRMP-2	O08553	7	4	103	62.3	5.95^h)^	0.51	0.350
1	43	5.1	MALDI	GFAP	P03995	39	13	79	49.9	5.28	0.85	0.823
2	41	5.02	MALDI	GFAP	P03995	48	18	155	49.9	5.28	1.33	0.628
3	40	4.85	MALDI	GFAP	P03995	25	9	66	49.9	5.28	8.93	0.056
4	27	7.4	LC-ESI	Glutathione S- transferase μ1	P10649	21	7	176	25.8	8.14	*+++*^i)^	ND
5	26	5.79	MALDI	Peroxiredoxin -6	O08709	44	8	121	24.7	5.72	1.58	0.389
6	24	5.02	LC-ESI	Peroxiredoxin -2	Q61171	38	8	372	21.6	5.2	0.90	0.850

a) The spots shown in Figure 1.

b) Mr_obs _and Mr_cal_, observed and theoretical Mr; pI_obs _and pI_cal_, observed and theoretical pI, respectively.

c) Probability-based Mowse score [52] calculated using MASCOT software [48].

d) Fold change of spot volume on 160 dai.

e) *p*-Value of Student's t-test. ND: not determined.

f) Mr_cal _and pI_cal _of non-phosphorylated CRMP-2^1-517^.

g) Mr_cal _and pI_cal _of non-phosphorylated CRMP-2^1-572^.

h) An approximate shift of -0.05 per phosphate moiety is expected [25,26].

i) Undetectable in the mock sample.

During the analysis, we were particularly interested in spots *a*, *b1*, *b2*, and *b3*, named according to their *acidic *or *basic *pI values (Figure 1). The intensity of spot *a *increased with the progression of the disease in ICR mice (Figure 1B) and C57BL/6J mice (not shown), whereas the intensity of spots *b1*, *b2*, and *b3 *decreased (Figure 1B). We also found that the intensity corresponding to spot *a *remained very weak in the mock control mice (Figure 1D). MS analysis demonstrated that spots *a*, *b1*, *b2*, and *b3 *invariably contained CRMP-2 (Table 1). This result was further confirmed by Western blot analysis with an anti-CRMP-2 antibody (C4G) (Figure 1E). The antibody revealed several additional spots undetectable by SYPRO Ruby staining due to their low abundance (compare Figures 1D and 1E). These spots appeared to reflect the diverse levels of phosphorylation because each spot was separated by an almost constant pI value that was indicative of protein phosphorylation [25,26], and because treatment with λ-phosphatase (λ-PPase) [27] prior to the 2-DE/Western blot analysis eliminated the spots in the acidic region (Figure 1E, top and bottom). Spot *b1 *had an observed molecular mass (Mr_obs_) of 61 kDa and pI (pI_obs_) of 5.77 (Table 1) that were close to the theoretical Mr (62,278 Da) and pI (5.95) of the non-phosphorylated full-length CRMP-2. On the other hand, spot *a *had an Mr_obs _of approximately 56 kDa and a pI_obs _of 5.45 (Table 1), and the λ-PPase treatment did not affect its electrophoretic mobility (compare Figure 1E, top and bottom).

### Structural elucidation of the 56-kDa form of CRMP-2 by LC-ESI-MS/MS

**Figure 2:**
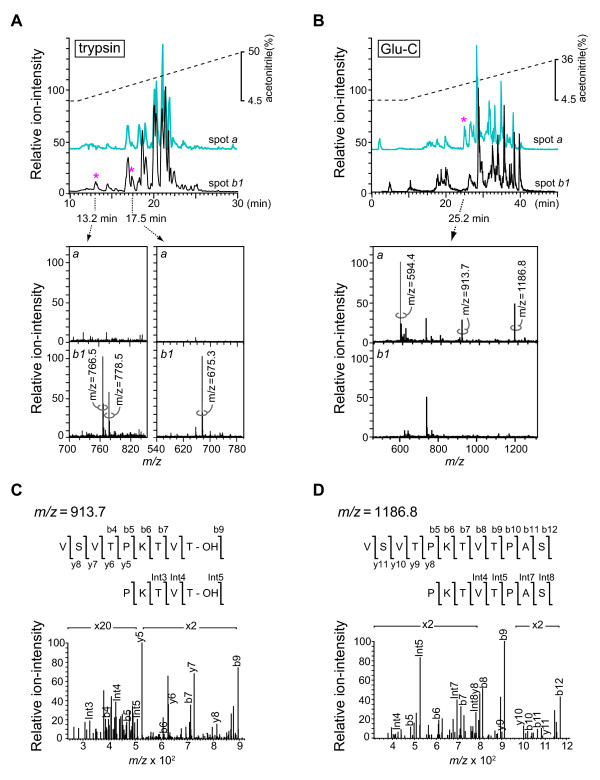
**Structural analysis of CRMP-2**. (A) Total ion chromatograms of tryptic digests derived from spots *a *(blue) and *b1 *(black). The peaks with asterisks were detectable in the digest of spot *b1*, but not spot *a*. Lower panels show the MS spectra of the fractions eluted at 13.2 min and 17.5 min, and the amino acid sequences determined by LC-ESI-MS/MS. (B) Total ion chromatograms from the Glu-C digest of spots *a *(blue) and *b1 *(black). The peak with an asterisk was detectable in the digests of spot *a*, but not spot *b1*. Lower panels show the MS spectra of the fraction eluted at 25.2 min, and the determined amino acid sequences. (C) LC-ESI-MS/MS spectrum and the sequence of the parental ion of m/z = 913.7. This parental ion was the b9 ion with a loss of a hydroxyl (-OH) group. The gain of the detector was modulated as indicated (× 2 or × 20 amplification) to optimize data acquisition. (D) MS/MS spectrum and the sequence of the parental ion of m/z = 1186.8.

**Figure 3:**
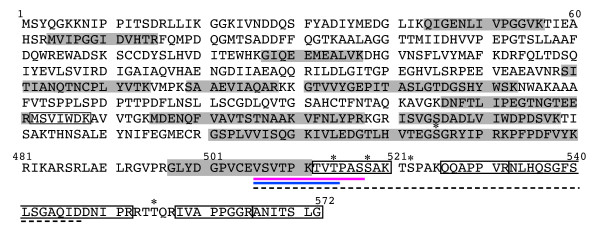
**Summary of the LC/MS analysis**. Gray: detected in the tryptic digests of both spots *a *and *b1*; white box: detected in the tryptic digest of spot *b1*; red underline: detected in the Glu-C digest of spot *a *([M+H]^+ ^= 1186.8); blue underline: detected in the Glu-C digest of spot *a *([M+H]^+ ^= 913.7); broken underline: expected to be obtained by the Glu-C digestion of full-length CRMP-2; ∗: the potential phosphorylation sites. The sequence is from UniProtKB; O08553.

To elucidate the structure of CRMP-2 in spots *a *(56 kDa) and *b1 *(61 kDa), we compared the tryptic peptides derived from the two spots by LC-ESI-MS/MS (Figure 2). In addition to the peptides detected in both digests (Figure 3, shown in grey), spot *b1 *yielded C-terminal peptides (Figure 3, indicated by boxes) including Ile^558^-Arg^565 ^([M+H]^+ ^= 766.5), Glu^526^-Arg^532 ^([M+H]^+ ^= 778.5) (Figure 2A, an asterisk at 13.2 min), and Ala^566^-Gly^572 ^([M+H]^+ ^= 675.3) (Figure 2A, an asterisk at 17.5 min). Recovery of these C-terminal peptides, together with the Mr_obs _(61 kDa), indicated that CRMP-2 in spot *b1 *was the full-length form. Conversely, the digest derived from spot *a *did not yield these C-terminal peptides (Figure 3). We then digested CRMP-2 in spots *a *and *b1 *using endoproteinase Glu-C (Glu-C) (Figure 2B). Full-length form of CRMP-2 is expected to release the C-terminal fragment Val^506^-Asp^547 ^(broken line in Figure 3) on digestion with Glu-C. However, this peptide was not detected in the digest of spot *a*; instead, the digest of spot *a *but not spot *b *gave rise to a unique peak in the chromatogram (an asterisk at 25.2 min in Figure 2B). MS analysis of this peak fraction detected three ion peaks at *m/z *values of 594.4, 913.7, and 1186.8 (Figure 2B). Subsequent LC-ESI-MS/MS analysis determined that the ion peaks at *m/z *1186.8 ([M+H]^+^) and 594.4 ([M+2H]^2+^) were derived from the peptide Val^506^-Ser^517 ^(Figure 2D), while the ion peak at *m/z *= 913.7 ([M+H]^+^) was ascribed to a b-type ion of Val^506^-Thr^514 ^(Figure 2C). This b-type ion may be produced by the fragmentation of the parental peptide Val^506^SerValThrProLysThrValThr^514^Pro^515^AlaSer^517 ^between the Thr^514^-Pro^515 ^bond, such a fragmentation between -X^n^--Pro^n+1^- to release b_n _and y_*N*-n _ions (Gly and Pro are unfavorable for X, *N *is the total number of amino acids) being observed in tandem MS [28]. Because Glu-C hydrolyzes Glu-X and Asp-X bonds, the presence of a Ser residue at the C-terminus strongly suggested that the peptides were originally at the C-terminus of CRMP-2 in spot *a*. In support of this, the values of Mr_obs _and pI_obs _of spot *a *were in good accord with the corresponding theoretical values for non-phosphorylated CRMP-2^1-517 ^(Table 1). Therefore, we concluded that the 56-kDa CRMP-2 in spot *a *was truncated at Ser^517^, and designated this shortened form CRMP-2-ΔC.

### The ratio of CRMP-2-ΔC to full-length CRMP-2 during the progression of prion disease

**Figure 4:**
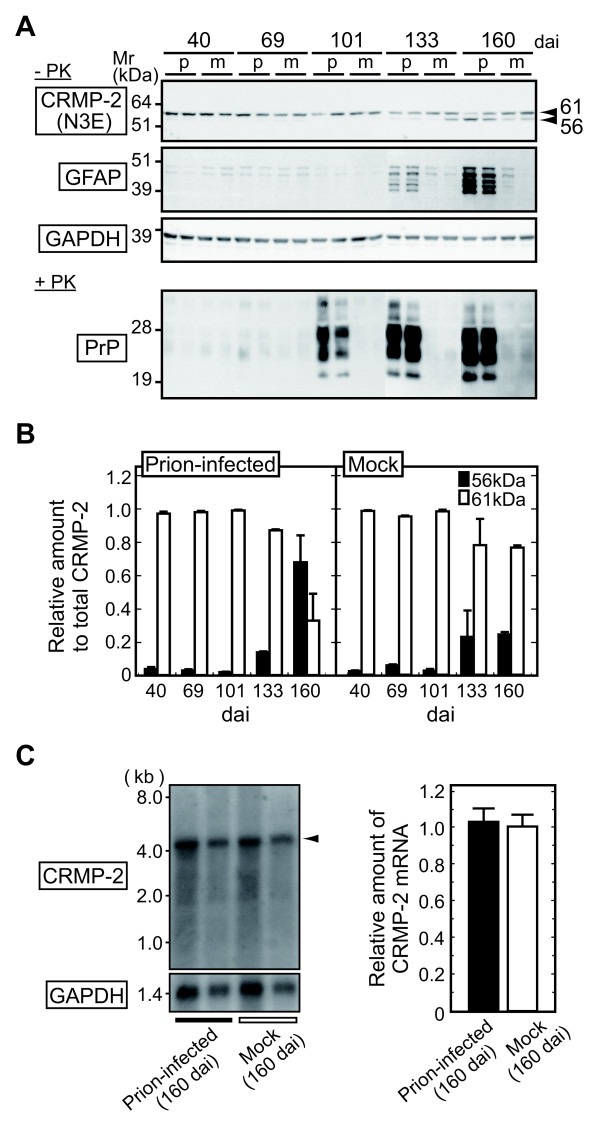
**CRMP-2-ΔC in the brains of C57BL/6J mice during the progression of prion disease**. (A) Western blot analysis for CRMP-2, GFAP and GAPDH. The accumulation of PK-resistant PrP^Sc ^is shown at the bottom. p: prion-infected; m: mock controls. The ladders of GFAP bands are possibly due to post-translational cleavage [24] or alternative splicing [51]. (B) The amounts of 61-kDa and 56-kDa CRMP-2 relative to the total amount of CRMP-2 as determined from the signal intensity of the bands in panel (A). Data are means ± SEM (n = 2). (C) Northern blot analysis for prion-infected and mock-infected mice sacrificed at 160 dai (n = 2, respectively). The arrowhead indicates the CRMP-2 transcript. The ratios of signal intensities of the bands of CRMP-2 mRNA to that of GAPDH mRNA are shown after normalization to the ratio obtained from the control mice as 1.00 (means ± SEM).

Completion of the structural analysis of CRMP-2-ΔC enabled us to use the N3E antibody to evaluate the relative amounts of CRMP-2-ΔC (56 κDa) and the full-length form (61 κDa). Since the full-length CRMP-2 and CRMP-2-ΔC both contain the epitope for the N3E antibody which recognizes the amino acid sequence 142-194 of CRMP-2 [29], the antibody should give comparable signal intensities of the two forms in Western blot analysis. We observed that the ratio of the amount of CRMP-2-ΔC (56 kDa) to the total amount of CRMP-2 (56 kDa + 61 kDa) was negligible at 40, 69, and 101 dai, and increased slightly at 133 dai in the prion-infected mice and the mock-infected controls (Figures 4A and 4B). In the controls sacrificed at 160 dai, the ratio increased in a narrow range and did not exceed 0.3 (Figure 4B, right). By contrast, in the prion-infected mice sacrificed at 160 dai when the expression of GFAP was up-regulated (Figure 4A), CRMP-2-ΔC increased moderately whereas the full-length form decreased. As a result, the amounts of CRMP-2-ΔC became comparable to those observed for the full-length form of CRMP-2 (Figure 4B, left). This change of CRMP-2-ΔC in the late stages of the disease was further confirmed by the examination of an additional number of specimens (Additional file 1). This modest but statistically significant change appeared to account for the focal distribution of lesions in the brain [1-3].

### Analysis of the CRMP-2 transcript

Two variant forms of CRMP-2, named CRMP-2A and -2B, were previously identified that had different amino terminal regions due to the alternative usage of two first coding exons [30-33]. Therefore, we examined if the occurrence of CRMP-2-ΔC was due to alternative splicing or post-translational processing. Using Northern blot analysis, a single 4.5-kb band was invariably detected in the prion-infected mice (n = 2) and the mock controls (n = 2) sacrificed at 160 dai (Figure 4C). This size was substantially the same as for the previously reported CRMP-2 gene transcript [17,19]; accordingly, we conceived that CRMP-2-ΔC was not produced by alternative splicing, but by post-translational proteolytic cleavage. It should be noted that densitometric analysis of the bands did not show significant difference in the mRNA levels of CRMP-2 between the prion-infected and the control mice after normalization to the amount of glyceraldehyde-3-phosphate dehydrogenase (GAPDH) mRNA (Figure 4C, right). In a further analysis by quantitative RT-PCR to compare the mRNA levels of CRMP-2 in the two groups of mice in detail, we again did not find a statistical difference in the level of CRMP-2 mRNA between the prion-infected mice and the mock controls (Additional file 2).

### Effects of the overexpression of CRMP-2-ΔC on neurons *in vitro*

**Figure 5:**
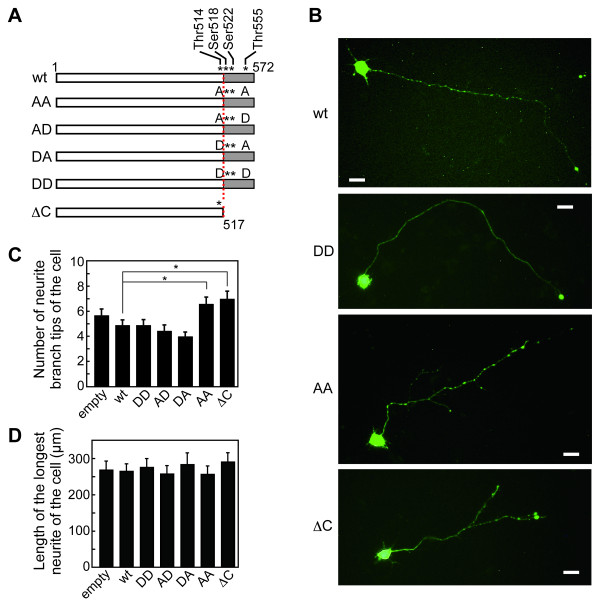
**Effect of CRMP-2-ΔC on primary cultured neurons**. (A) Diagram of the cDNA constructs. The asterisks show the potential phosphorylation sites in the C-terminal region. (B) Representative images of the neurons at 4-DIV. Bar; 20 μm. (C) Numbers of neurite branch tips longer than 10 μm in individual cells at 4-DIV. Data are means ± SEM from at least 24 cells. ^∗^Statistical difference determined by Student's t-test (*P*0.05). (D) Lengths of the longest neurites of the cells at 4-DIV. Data are means ± SEM of the lengths from at least 24 cells.

Considering the increasing ratio of CRMP-2-ΔC to full-length CRMP-2 in the late stages of prion disease in our model (Figure 4), we asked whether CRMP-2-ΔC would affect the morphology of the neurites. Plasmids encoding full-length CRMP-2 (named CRMP-2-wt), CRMP-2-ΔC, or phosphorylated or non-phosphorylated mimics named CRMP-2/DD, /AD, /DA, and /AA, in which the C-terminal potential phosphorylation sites Thr^514 ^and Thr^555 ^[20] were mutagenized to Asp or Ala (Figure 5A), were introduced into murine embryonic cerebral neurons on the first day of culture *in vitro *(0-DIV). As the transfection efficacy of the neurons in primary culture was rather low, we co-transfected a plasmid encoding green fluorescent protein (GFP) to identify the transfected cells. The cells positive for GFP-fluorescence were examined at 4-DIV for neurites longer than 10 μm, and we found that the cells transfected with CRMP-2-ΔC developed 1.2-fold more neurite tips than the cells transfected with the empty vector (Figures 5B and 5C). An enhanced level of branching of the neurite tips was also observed in the cells expressing the non-phosphorylated mimic CRMP-2/AA (Figures 5B and 5C). Conversely, the cells transfected with CRMP-2-wt exhibited slightly fewer neurite branch tips than the cells transfected with the empty vector, similar to the cells transfected with CRMP-2/DD (Figures 5B and 5C). This was presumably because cellular kinases phosphorylated CRMP-2-wt that, in turn, acted against the development of neurite tips. In contrast to the range of variation in branching, the length of the longest neurites did not show a significant difference and were in the range of 269.8 ± 10.1 μm (Figure 5D).

## Discussion

CRMPs are cytosolic phosphoproteins abundantly expressed in the developing brain [16-20,31], and in some neurons and oligodendrocytes in the adult brain [17,19,32,33]. Among the five known isoforms (CRMP-1 to -5), CRMP-2 was originally identified as a mediator of the collapse of growth cones by semaphorin [16], and later shown to be involved in the outgrowth of axons and the induction of neuronal polarity [20] in developing neurons. Further studies also showed different splicing patterns of its mRNA, giving rise to two variant forms named CRMP-2A and -2B [30]. CRMP-2A (theoretical Mr of 75 kDa) has a long N-terminal sequence, and induces oriented microtubule patterns in cultured fibroblasts [30]. On the other hand, the shorter variant CRMP-2B (theoretical Mr of 62 kDa) corresponds to the originally identified CRMP-2 protein, and favors disoriented microtubule patterns in the fibroblasts [30]. Thus, the two variants seem to display different, or even opposite functional properties [30,32]. Intriguingly, Western blot analysis of the brain of mouse [32] and rat [33] showed that CRMP-2A is mainly expressed in the developing brain while CRMP-2B is predominantly expressed in the adult brain. The temporal expression pattern of the two forms, together with their distinct functions, suggest that CRMP-2 plays roles more than the axonal outgrowth and the induction of neuronal polarity.

Not only in the physiological neuronal processes, the importance of CRMP-2 in the adult brain under nonphysiological conditions has also been implied recently in animal models of neurodegenerative disorders including traumatic brain injury and cerebral ischemia (see below). Here, we performed a proteomic analysis of the brains of mice infected with scrapie prion, and found a C-terminally truncated form of CRMP-2 (CRMP-2-ΔC) in the late pathological stages of the disease. To date, truncated forms of CRMP-2 have been identified in cultured neurons exposed to N-methyl-D-aspartic acid [34,35] or depleted of nerve growth factor [36], in models of traumatic brain injury [35,37], ischemia [38], and sciatic nerve injury [39], and in the developing mouse brain [31]. Although the precise cleavage sites of CRMP-2 in these studies remain undetermined, sequence-specific antibodies [34,35] or peptide mapping in MS analyses [31,36,39] mapped them to the C-terminal region. In addition, some studies ascribed proteolytic cleavage by calpain to the production of the truncated forms of CRMP-2 [34-36]. We demonstrated that the production of CRMP-2-ΔC was not due to the alternative splicing of its mRNA, the known splicing responsible for the production of CRMP-2A and -2B [30]. Rather, judging from its Mr_obs_, pI_obs _and the truncation site, it is most conceivable that CRMP-2-ΔC was derived from proteolytic cleavage of CRMP-2B (referred to as the full-length form of CRMP-2 in the present study) which is predominantly expressed in the adult brain [32,33]. We speculate that calpain is likely to be involved in its generation, since the truncation site of CRMP-2-ΔC (P_4 _= Thr, P_3 _= Pro, P_1_' = Ser, and P_2_' = Ala) was in modest agreement with the amino acid sequences preferred for cleavage by calpain [40,41]. In this regard, an increased level of μ-calpain was reported in the hippocampus of prion-infected mice [24]. It should be noted that a recent quantitative RT-PCR analysis of the brains of C57BL/6 mice challenged with bovine spongiform encephalopathy prion showed an increased level of CRMP-2 mRNA approximately to 140% of that in the control mice in the mid-stage of the disease, and its decrease to 54% in the terminal stage [42]. We have shown the level of CRMP-2 mRNA did not differ significantly between the prion-infected and the control mice in the late phase of the disease (Figure 4C, Additional file 2). The reason for this discrepancy in the decreased level of CRMP-2 mRNA in the later stages of the disease remains unknown at the moment. It might be due to the difference in the prion strains, or the difference in the pathological severity at the timing of the specimen sampling. The truncation of CRMP-2 was not mentioned in this transcriptional study [42].

Lines of evidence to date indicate that the sequential phosphorylation of CRMP-2 at Ser^522^, Thr^509^, Thr^514 ^and Ser ^518 ^by Cdk5 and GSK-3β is essential for the regulation of CRMP-2 activity [20,29]. These regulatory sites are, however, partially ablated in CRMP-2-ΔC. In fact, the unchanged pI value of CRMP-2-ΔC after incubation with λ-PPase (Figure 1E), and the absence of a neutral loss of H_3_PO_4 _(Δ*m/z *= -98) in the MS analysis of CRMP-2-ΔC (data not shown), strongly indicated that it was not phosphorylated. Thus, the augmentation in the development of neurite branch tips of cultured neurons by the overexpression of CRMP-2-ΔC may be explained by the lack of the regulatory phosphorylation sites. Alternatively, it is recognized that CRMPs are assembled into homo- or hetero-tetramers. Interestingly, while the structure of the recombinant CRMP-2^13-490 ^(i.e., lacking the C-terminal region) was indeed in a tetrameric form, as determined using crystallographic analysis, it behaved like a trimer when analyzed using gel filtration [43]. This was interpreted to indicate a rapid equilibrium between the tetrameric and the dimeric states of CRMP-2^13-490 ^[43]. If so, it is conceivable that CRMP-2-ΔC has a reduced ability to undergo oligomerization. Such a defect in oligomerization into the homomeric and/or heteromeric state would impair the physiological function of CRMP-2. In the adult brain, CRMP-2B is the predominant form of CRMP-2, and mainly localizes in dendrites [32]. Considering that hamster [2] and murine [3] models of scrapie showed neuronal dendritic atrophy before ultimate neuronal cell death, it is interesting to surmise that CRMP-2-ΔC might be involved in such a neurodegenerative process.

## Conclusions

To explore the molecular neuropathology of prion diseases, we conducted a proteomic analysis of a murine model of prion diseases. In addition to the quantitative changes of GFAP, glutathione S-transferase-μ1 and peroxiredoxins during the progression of the disease in the brain of ICR and C57BL/6J mice, we identified a unique truncation of CRMP-2. Detailing the relevance of CRMP-2-ΔC to the morphological abnormalities of degenerating axon terminals/dendrites observed in models of scrapie [1-3] awaits further study. Nevertheless, considering that several forms of truncated CRMP-2, if not identical to CRMP-2-ΔC, were found in models of neurodegenerative disorders [35-39], the results of the present study should provide clues to the molecular neuropathology of prion diseases compared with other neurodegenerative disorders.

## Methods

### Mice, prion inoculation, and brain homogenate

Female C57BL/6J and ICR mice (6 weeks old) were purchased from Charles River Laboratories (Yokohama, Japan) and CLEA Inc. (Meguro, Japan), respectively. The mice were inoculated with 25 μL of a 0.25% (w/v) brain homogenate in phosphate-buffered saline (PBS; JRH Biosciences, Lenexa, USA) prepared from terminally ill ICR mice infected with mouse-adapted scrapie prion (Obihiro-I strain) [21]. As mock-infected controls, mice were injected with 25 μL of a 0.25% brain homogenate (w/v in PBS) from healthy mice. Experiments were carried out in compliance with the biosafety regulations and the guidelines for laboratory animal care of the National Institute of Infectious Diseases. Individual brains were homogenized in 4-volumes (w/v) of chilled PBS using a Physcotron homogenizer (Microtec Co., Ltd., Funabashi, Japan), and centrifuged at 100,000 × *g *for 1 h at 4°C with a TLA-55 rotor (Beckman Coulter, Inc., Fullerton, USA) to obtain the supernatant (soluble fraction) and the pellet (membranous fraction). Protein concentrations were determined using a BCA protein assay kit (Pierce, Rockford, USA).

### 2-DE

The samples were prepared by adjusting the soluble fraction of the brain homogenate to a total of 400 μg protein/mL in 9 M Urea, 4% CHAPS, 65 mM DTT, and 0.5% IPG buffer 3-10 (GE Healthcare, Uppsala, Sweden). Immobiline DryStrips for isoelectric focusing (IEF; pH 3-10 NL, 13 cm, GE Healthcare) were rehydrated at 20°C overnight by soaking in 250 μL of the sample solution (a total of 100 μg of protein), then IEF was carried out using a linear gradient from 0-1000 V in 2 h, followed by 8000 V for 6 h. After IEF, the strips were equilibrated with 50 mM Tris-HCl (pH 8.8), 6 M urea, 2% SDS, 20 mM DTT, 30% glycerol, and 0.03% bromophenol blue for 15 min, and overlaid on 12.5% polyacrylamide slab gels for SDS-PAGE. Proteins were stained with CBB R-250 or SYPRO Ruby (Invitrogen, Carlsbad, USA), and gel images were captured using a LAS-1000 plus lumino-image analyzer (Fuji Photo Film, Tokyo, Japan). Software PDQuest, version 7.3 (Bio-Rad Laboratories, Hercules, USA) was used for the image analysis. At least one duplicate set of mice was analyzed for each specified dai. Mr and pI were calibrated using the spots of the following proteins: calmodulin (16.7 κDa, pI 4.1), γ-enolase (47.2, 5.0), serum albumin (65.9, 5.5), peroxiredoxin 6 (24.7, 5.72), carbonic anhydorase II (28.9, 6.5), and aconitase (82.5, 7.4). The signal intensity of each spot was normalized to that of β-actin for statistical analysis. In the dephosphorylation analysis, the samples were incubated with λ-PPase (New England Biolabs, Inc., Beverly, USA) at 30°C for 15 min prior to 2-DE.

### Western blotting

The anti-CRMP-2 antibodies C4G (epitope; CRMP-2^480-528^) and N3E (epitope; CRMP-2^142-194^) [29] were purchased from Immuno-Biological Laboratories Co., Ltd. (Takasaki, Japan). The other antibodies used were anti-GFAP (DAKO, Glostrup, Denmark), anti-GAPDH from (Abcam plc., Cambridge, UK), HRP-labeled anti-mouse IgG (TrueBlot) (eBioscience Inc., San Diego, USA), and HRP-labeled anti-rabbit IgG F(ab')_2 _(GE Healthcare). The ECL Plus reagent (GE Healthcare) and a LAS-3000mini lumino-image analyzer (Fuji Photo Film) were used for detection. Signal intensity was quantified using Image Gauge software (Fuji Photo Film). For the analysis of PrP^Sc^, the membranous fraction (a total of 10 μg proteins) in 30 μL of 0.5% sarkosyl, 25 mM Tris-HCl, and 50 mM NaCl (pH 7.5) was incubated with PK (final concentration, 0.1 μg/μL) at 37°C for 120 min. The digestion was stopped by the addition of 4-volumes of 10 mM phenylmethylsulfonyl fluoride in methanol, and the mixture was centrifuged at 20,000 × *g *for 30 min at 4°C to recover PrP^Sc ^as a precipitate. The anti-PrP monoclonal antibody 44B-1 [44] was a gift from Prof. M. Horiuchi (Hokkaido University, Japan).

### MS and protein identification

Pieces of the 2-DE gel were rinsed with 100 mM ammonium bicarbonate, and incubated with 10 mM DTT at 56°C for 45 min. Then, protein in the gel was reacted with 55 mM iodoacetamide at room temperature for 45 min, and subjected to in-gel digestion [45,46] with trypsin (sequencing grade; Promega, Madison, USA) or Glu-C (EC 3.4.21.19; Roche Applied Science, Basel, Switzerland). For the peptide-mass fingerprint analysis, MS data for the tryptic digests obtained with a Voyager-DE STR spectrometer (Applied Biosystems, Foster City, USA) were examined in the NCBInr.fasta protein database using MS-Fit [47] and Mascot/version 2.1 (Matrix Science, Boston, USA) software [48]. For the amino acid sequence analysis of CRMP-2, the digests were applied to a MAGIC2002 HPLC system (Michrom Bioresources, Inc., Auburn, USA) equipped with a capillary column (Inertsil ODS 3 μm, 0.1 mm i.d. × 50 mm, GL Science Inc., Shinjuku, Japan). Elution was performed using (A) H_2_O/acetonitrile/formic acid = 98/2/0.1 (v/v/v) and (B) H_2_O/acetonitrile/formic acid = 10/90/0.1 (v/v/v), with a gradient from A/B = 95/5 (v/v) to 45/55 (v/v) at a flow rate of 400 nL/min. The eluate was introduced into an LCQ Deca XP spectrometer (Thermo Fisher Scientific Inc., Waltham, USA) using a nano-ESI interface (AMR Inc., Meguro, Japan) and a MonoSpray FS monolithic emitter (GL Sciences). The data were collected at an interval of three tandem spectra per spectrum in the data-dependent-scan mode, and analyzed using BioWorks software (Thermo Fisher Scientific Inc., version 3.1) and by manual inspection. The theoretical Mr and pI values were calculated using Compute pI/MW [49] and Scansite [50] software.

### Northern blotting

RNA was prepared from the whole brains of C57BL/6J mice sacrificed at 160 dai using the SV Total RNA Isolation System (Promega), and stored at -80°C in RNA*secure *reagent (Applied Biosystems) until use. Electrophoresis was carried out with 1 μg of RNA using 1.0% agarose-formaldehyde gel in 40 mM MOPS-NaOH, 10 mM sodium acetate, and 1 mM EDTA (pH 7.0). After the transfer of the RNA to a positively charged-nylon membrane (Roche Applied Science), the membrane was incubated with digoxigenin-labeled antisense RNA probes for CRMP-2 (complementary to the open reading frame (ORF) from +340 to +1551; corresponding to Thr^114^-Ser^517^) or GAPDH (the ORF of from +330 to +878) at 68°C overnight in Ambion ULTRAhyb hybridization buffer (Applied Biosystems). The bound probes were detected using an alkaline phosphatase-labeled anti-digoxigenin antibody (Roche Applied Science) and CDP-Star chemiluminescence reagent (New England Biolabs, Inc.), with X-ray films (Fuji Photo Film) and a LAS-3000mini lumino-image analyzer (Fuji Photo Film). Signal intensity was quantified using Image Gauge software (Fuji Photo Film).

### Plasmids

Full-length cDNA of mouse CRMP-2-wt was obtained from a C57BL/6J mouse brain cDNA library (Invitrogen) by PCR using the following primers: 5'- GGAATTCGAGATGTCTTATCAGGGGAAGAAAAATATTCCA-3' and 5'- ATAAGAATGCGGCCGCTTTAGCCCAGGCTGGTGATGTTGG-3'	(the	underlined sequences are for the generation of *Eco*RI and *Not*I sites). CRMP-2-ΔC cDNA (CRMP-2^1-517^) was generated using the reverse primer 5'- ATAAGAATGCGGCCGCTTATGAGGCTGGAGTCACC-3' (the underlined sequence is for the introduction of a *Not*I site). The cDNAs were ligated into the pCIneo mammalian expression vector (Promega) between the *Eco*RI and *Not*I sites. Site-directed mutagenesis was carried out using the following primers: 5'- GTGACGCCCAAGACGGTGGCGCCAGCCTCATCAGCTAAG-3' and 5'- CTTAGCTGATGAGGCTGGCGCCACCGTCTTGGGCGTCAC-3' for T514A; 5'- GTGACGCCCAAGACGGTGGATCCAGCCTCATCAGCTAAG-3' and 5'- CTTAGCTGATGAGGCTGGATCCACCGTCTTGGGCGTCAC-3' for T514D; 5'- CATTCCCCGCCGCACCGCCCAGCGCATCGTGG-3' and 5'- CCACGATGCGCTGGGCGGTGCGGCGGGGAATG-3' for T555A; and 5'- CATTCCCCGCCGCACTGATCAGCGCATCGTGG-3' and 5'- CCACGATGCGCTGATCAGTGCGGCGGGGAATG-3' for T555D. The constructs were verified using DNA sequencing.

### Cell culture, transfection, and microscopy

Cerebral cortical neurons from E15 ICR mice (Nerve-cell Culture System; Sumitomo Bakelite Co., Ltd., Shinagawa, Japan) were plated on poly-L-lysine-coated plastic dishes (Sumitomo Bakelite Co., Ltd.), and maintained in Neurobasal medium (Invitrogen-GIBCO) with B27 (Invitrogen-GIBCO) and 0.5 mM glutamine according to the manufacturer's instructions. Arabinocytidine (2.5 μM) was added to the medium to suppress the growth of glia. The pCIneo vectors encoding CRMP-2 or its mutants (1 μg) were mixed with pEGFP-N1 encoding GFP (0.5 μg; Clontech Laboratories Inc., Mountain View, USA), and the mixture was introduced into cells using the NeuroPORTER transfection reagent (Genlantis, San Diego, USA) at the time of plating (0-DIV). The neurons were fixed at 4-DIV using BD Cytofix (BD Biosciences, Franklin Lakes, USA) for 15 min at room temperature, and observed under a BIOZERO BZ-8000 microscope (Keyence, Osaka, Japan) with a software BZ analyzer (Keyence) for counting the number of neurite tips and measuring the length of neurites.

## List of abbreviations

**CRMP-2**: collapsin response mediator protein-2; **2-DE**: two-dimensional electrophoresis; **dai**: days after inoculation; **DIV**: day of culture *in vitro*; **GAPDH**: glyceraldehyde-3-phosphate dehydrogenase; **GFAP**: glial fibrillary acidic protein; **GFP**: green fluorescent protein; **Glu-C**: endoproteinase Glu-C; **IEF: **isoelectric focusing; **λ-PPase**: lambda phosphatase; **LC-ESI-MS/MS**: liquid chromatography-electrospray ionization-tandem mass spectrometry; **MALDI**: matrix-assisted laser desorption/ionization; **Mr**: molecular mass; **Mr_obs_**: observed Mr; **MS**: mass spectrometry; **ORF**: open reading frame; **PBS: **phosphate-buffered saline; **pI_obs_**: observed pI; PK: proteinase K; **PrP**: prion protein; **PrP^C^**: cellular prion protein; **PrP^Sc^**: disease-associated prion protein

## Competing interests

The authors declare that they have no competing interests.

## Authors' contributions

**FSO **performed the experiments, data analysis and protein identification, and wrote the manuscript. **YY **took care of the mice, collected tissue samples, provided experimental devices, and took part in the experimental design. **HH **took care of the mice and collected tissue samples. **MT **helped perform the cell culture experiments. **MN **and **KH **took part in the experimental design. **KH **performed a Northern blotting analysis, participated in the experimental design, data analysis, and wrote the manuscript. All authors read and approved the final manuscript.

## Supplementary Material

Additional file 1 **CRMP-2-ΔC in the brains of C57BL/6J mice in the late stages of the disease**. A supplementary data of Western blot analysis for detection of CRMP-2-ΔC in additional individuals of prion-infected and mock control mice. Click here for file

Additional file 2 **Relative levels of CRMP-2 mRNA determined by quantitative RT-PCR**. The mRNA levels of CRMP-2 did not show significant difference between the prion-infected and mock control mice in quantitative RT-PCR analysis. Click here for file
