# Supplementary material for: Identification and structural analysis of C-terminally truncated collapsin response mediator protein-2 in a murine model of prion diseases
Source: Proteome Sci. 2010 Oct 20;8:53. doi: 10.1186/1477-5956-8-53 (PMC2978134; doi:10.1186/1477-5956-8-53)
Supplement: Additional file 1 — CRMP-2-ΔC in the brains of C57BL/6J mice in the late stages of the disease. A supplementary data of Western blot analysis for detection of CRMP-2-ΔC in additional individuals of prion-infected and mock control mice. [file 1477-5956-8-53-S1.PDF]

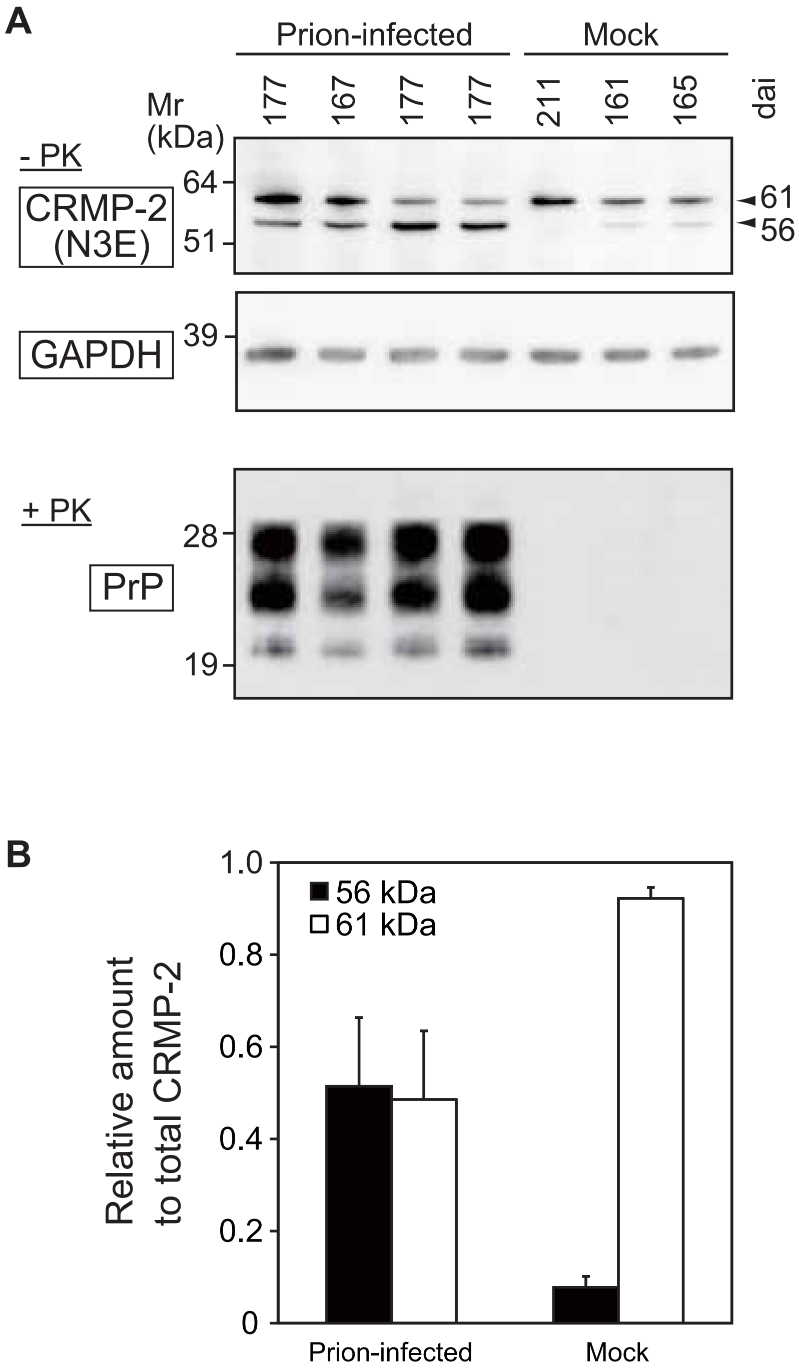

Additional File 1 CRMP-2-ΔC in the brains of C57BL/6J mice in the late stages of the disease.
(A) Western blot analysis for CRMP-2, GAPDH, and PK-resistant PrP^{Sc}. The numbers above the lane indicated the time of days after inoculation. (B) The amount of 61-kDa and 56-kDa CRMP-2 relative to the total amount of CRMP-2 as quantified from the signal intensity of the bands in panel (A). Data are meas ± SEM (n = 4 for prion infected; n =3 for mock).
