# Supplementary material for: Identification and structural analysis of C-terminally truncated collapsin response mediator protein-2 in a murine model of prion diseases
Source: Proteome Sci. 2010 Oct 20;8:53. doi: 10.1186/1477-5956-8-53 (PMC2978134; doi:10.1186/1477-5956-8-53)
Supplement: Additional file 2 — Relative levels of CRMP-2 mRNA determined by quantitative RT-PCR. The mRNA levels of CRMP-2 did not show significant difference between the prion-infected and mock control mice in quantitative RT-PCR analysis. [file 1477-5956-8-53-S2.PDF]

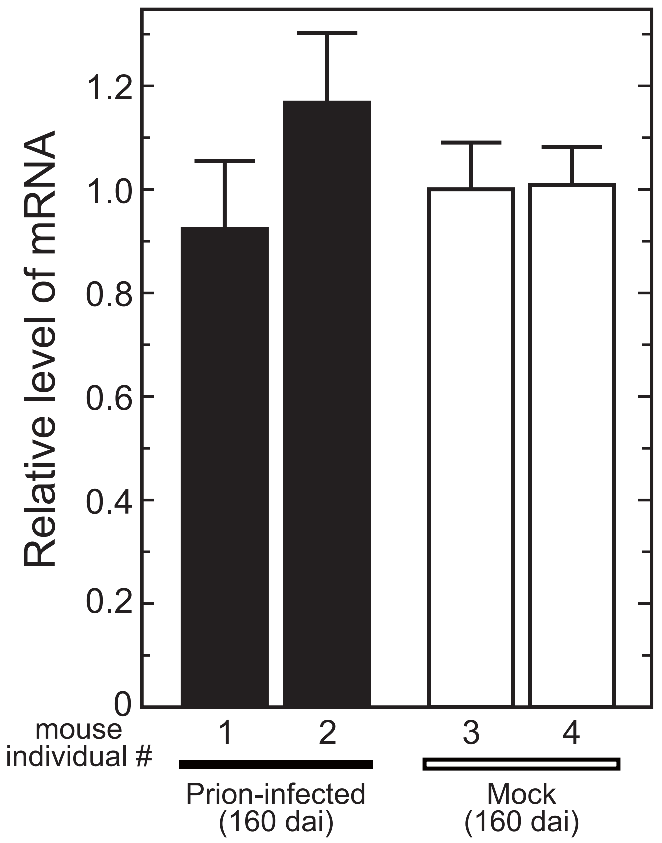

Additional File 2 Relative levels of CRMP-2 mRNA determined by quantitative RT-PCR.

Quantitative RT-PCR (qRT-PCR) was carried out using 0.3 μg of total RNA from the whole brain of C57BL/6J mice sacrificed at 160 dai, SuperScript III Platinum SYBR Green one step qRT-PCR reagent (Invitrogen), the LightCycler system (Roche Applied Bioscience), and the following primers; CRMP-2-Fw; 5'-CGG GGT AAA CTC CTT CCT CGT GTA CGG GGT AAA CTC CTT CCT CGT GTA-3' (a sense oligonucleotide in spanning exon 3 and 4), CRMP-2-Rv; 5'-TGG TCT GGT TGG CAA TAG TGA TGG A-3' (an antisense oligonucleotide in exon 8), GAPDH-Fw; 5'-TGT GTC CGT CGT GGA TCT GA-3' (sense in exon 5), and GAPDH-Rv; 5'-TTG CTG TTG AAG TCG CAG GAG-3' (antisense in exon 6). The thermal cycle program for CRMP-2 mRNA was follows: at 50 ˚C for 8 min for reverse-transcription, followed by denature at 95 ˚C for 1min, then 50 cycles of PCR at 92 ˚C for 5 s, 60 ˚C for 5 s, and 72 ˚C for 10 s. The thermal cycle program for GAPDH mRNA was the same except that the reverse transcription was carried out at 50 ˚C for 6min, and the annealing step of the PCR was at 61 ˚C for 5 s. The data are means ± SEM of the ratio of the amount of CRMP-2 mRNA to that of GAPDH mRNA (three independent experiments in a triplicate for each individual), after normalization to the ratio obtained from the mouse-#3 (a mock control) as 1.00.
